# ﻿First record of the spider family Trechaleidae Simon, 1890 (Araneae) from China

**DOI:** 10.3897/zookeys.1203.124808

**Published:** 2024-05-30

**Authors:** Lu-Yu Wang, Yan-Nan Mu, Feng Zhang, Yuri M. Marusik, Zhi-Sheng Zhang

**Affiliations:** 1 Key Laboratory of Eco-environments in Three Gorges Reservoir Region (Ministry of Education), School of Life Sciences, Southwest University, Chongqing 400715, China Southwest University Chongqing China; 2 The Key Laboratory of Zoological Systematics and Application, Institute of Life Science and Green Development, College of Life Sciences, Hebei University, Baoding, Hebei 071002, China Hebei University Baoding China; 3 Institute for Biological Problems of the North RAS, Portovaya Str.18, Magadan 685000, Russia Institute for Biological Problems of the North RAS Magadan Russia; 4 Department of Zoology & Entomology, University of the Free State, Bloemfontein 9300, South Africa University of the Free State Bloemfontein South Africa; 5 Altai State University, Lenina Pr., 61, Barnaul, RF-656049, Russia Altai State University Barnaul Russia

**Keywords:** Description, morphology, new species, taxonomy, Xizang

## Abstract

The family Trechaleidae Simon, 1890 is reported for the first time from China, including one new species: *Shinobiuscona***sp. nov.** (♂♀). Morphological descriptions, photos and illustrations of the new species are provided. Taxonomic features of species belonging to the genus are briefly discussed. Photos of the female of *Shinobiusorientalis* (Yaginuma, 1967) are also presented to compare it with the new species.

## ﻿Introduction

The spider family Trechaleidae is relatively small, with 133 named species belonging to 17 genera (WSC 2024). Sixteen genera and 132 species are restricted to the Neotropical Realm, and only one monotypic genus, *Shinobius* Yaginuma, 1991 is known in the Palaearctic Realm (Japan) (WSC 2024).

While studying specimens collected from Xizang, China, we found two specimens of both sexes that are similar to *Shinobius* in somatic morphology and features of the male palp and epigyne. These specimens, observed in the field, construct funnel-shaped webs and carry egg sacs by spinnerets. The goal of this paper is to provide a detailed description of the new species and a brief discussion of the taxonomic position of the genus.

## ﻿Material and methods

All specimens are preserved in 75% ethanol and were examined, illustrated, photographed, and measured using a Leica M205A stereomicroscope equipped with a drawing tube, a Leica DFC450 camera, and LAS software (v. 4.6). Male palps and epigynes were examined and illustrated after they were dissected. Epigynes were cleared by immersing them in pancreatin (Álvarez-Padilla and Hormiga 2007). Eye sizes were measured as the maximum diameter. Leg measurements are shown as total length (femur, patella and tibia, metatarsus, tarsus). All measurements are in millimetres. The specimens examined here are deposited in the Collection of Spiders, School of Life Sciences, Southwest University, Chongqing, China (SWUC).

Comparative material: *Shinobiusorientalis*: 1♀ Japan, Ibaraki Pref., Sakuragawa, Hatori, 36°14'10.5"N, 140°05'58.1"E, 23.vi.2018, R. Kuwahara leg.

Abbreviations used in the text: ALE – anterior lateral eye; **AME** – anterior median eye; PLE – posterior lateral eye; **PME** – posterior median eye.

## ﻿Taxonomy

**Family Trechaleidae Simon, 1890**

### ﻿Genus Shinobius

Taxon classificationAnimaliaAraneaeTrechaleidae

Yaginuma, 1991

9ED5AA30-4D08-5CF6-9F76-BC56B1543ED3

#### Type species.

*Cispiusorientalis* Yaginuma, 1967.

#### Diagnosis.

*Shinobius* is similar to the South American genera *Rhoicinus* Simon, 1898 and *Barrisca* Chamberlin & Ivie, 1936, by the lack of the retrolateral tibial apophysis and having a very large subtegulum composing almost a half of the bulb. However, *Shinobius* can be separated from *Rhoicinus* and *Barrisca* by the cymbial tip shorter than the bulb and a strongly sclerotized posteroretrolateral part of the cymbium (vs. tip of cymbium longer than bulb, basal part of cymbium not modified) and by the presence of a median plate in the epigyne (vs. absent). *Shinobius* differs from other genera considered in the family by the lack of an extending retrolateral tibial apophysis.

#### Description.

Carapace brown. Eight eyes arranged in two rows, posterior row strongly protruding. Fovea longitudinal. Cervical groove indistinct, radial furrows distinct. Chelicerae yellow brown, with three promarginal and three retromarginal teeth. Endites and labium yellow brown, longer than wide. Sternum yellow brown, shield-shaped, with brown setae. Legs yellow brown, with black pigmentation. Leg formula: 4213. Opisthosoma oval. Dorsum yellow brown, with black brown markings. Venter yellowish-brown.

***Male palp***: tibia without extending retrolateral apophysis (RTA), but with strongly sclerotized kind of hood; cymbium droplet-shaped, with tip shorter than bulb, spines and claws present or absent; posteroretrolateral part strongly sclerotized (Cs, Fig. 3B). Subtegulum large, almost half of bulb, with anterior margin slanting; median apophysis (Ma) short, located on retrolateral half of bulb; conductor finger-shaped, longer than wide; embolus with oval-shaped base, filamentous, round bent at about right angle, tip located close to tip of median apophysis.

***Epigyne***: epigynal plate slightly wider than long; with a wide septum in type species and round in *S.cona* sp. nov.; fovea divided by septum; septum terminates near epigastral fold.

#### Composition.

*Shinobiuscona* sp. nov. and *S.orientalis* (Yaginuma, 1967).

#### Relationships.

*Shinobius* is the only genus of the family found far away from the rest of the genera which are distributed in the Neotropical Realm. *Shinobius* lacks a developed tibial apophysis (extending in from the tibia) but has instead a kind of hood with a strongly chitinized anterior margin lacking in other members of the family except for *Rhoicinus*. Based on this similarity and the shape of the bulb, Sierwald (1993) considered the two genera in a separate subfamily Rhoicinae Simon, 1898.

#### Distribution.

China (Xizang) and Japan (Fig. 4).

### ﻿ Shinobius cona sp. nov.

Taxon classificationAnimaliaAraneaeTrechaleidae

48E0DD1B-9FE4-56F9-8405-3E453B31B98A

https://zoobank.org/ADFEFF8E-E6C0-4652-AB32-7DB37D101BD8

**Figure 1.**
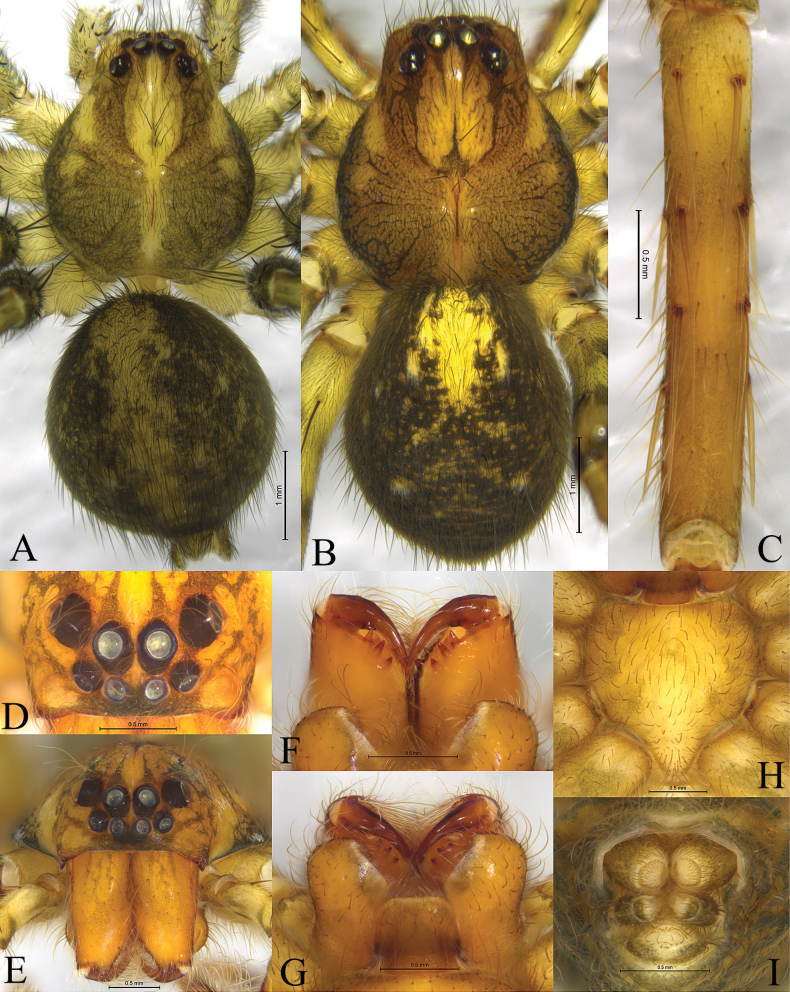
*Shinobiuscona* sp. nov. male holotype (**A**), female paratype (**B–I**) **A** male habitus, dorsal view **B** female habitus, dorsal view **C** tibia I, ventral view **D** eyes, dorsal view **E** eyes and chelicerae, front view **F** chelicerae, ventral view **G** chelicerae, endites and labium, ventral view **H** sternum, ventral view **I** spinneret, ventral view.

**Figure 2.**
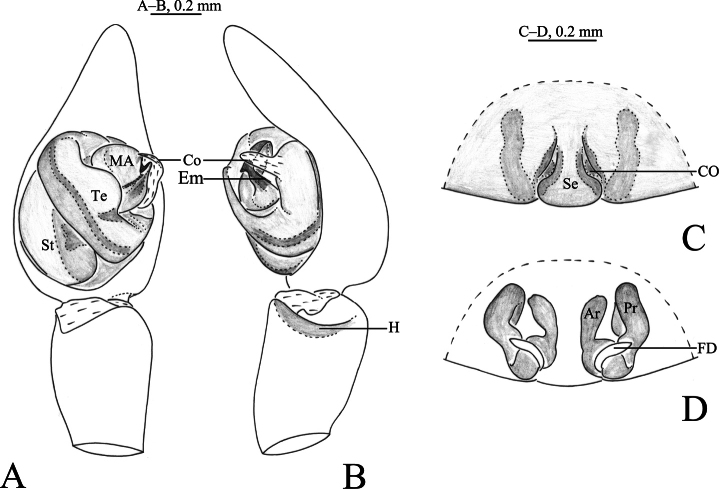
*Shinobiuscona* sp. nov. **A, B** holotype male **C, D** paratype female **A** left male palp, ventral view **B** same, retrolateral view **C** epigyne, ventral view **D** vulva, dorsal view. Abbreviations: Ar = anterior receptacle; CO = copulatory opening; Co = conductor; Em = embolus; FD = fertilization duct; MA = median apophysis; Pr = posterior receptacle; H = hood; Se = septum; St = subtegulum; Te = tegulum.

**Figure 3.**
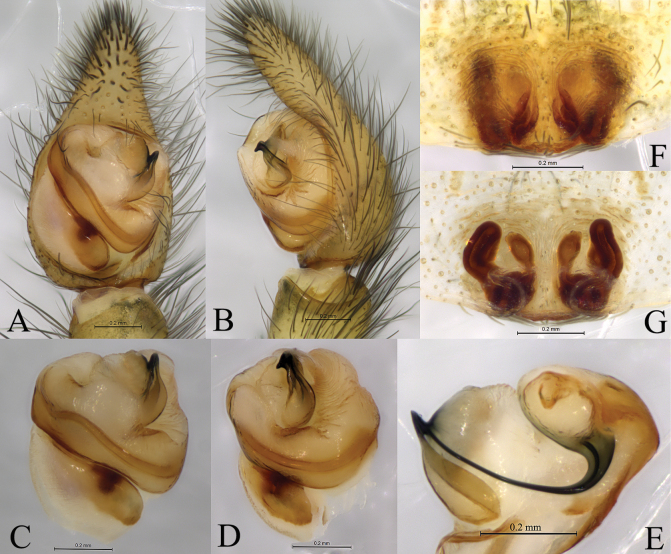
*Shinobiuscona* sp. nov. male holotype (**A–E**), female paratype (**F, G**) **A** left male palp, ventral view **B** same, retrolateral view **C** right male palp, bulb, ventral view (overturn) **D** same, retrolateral view (overturn) **E** right male palp, median apophysis and embolus, dorsal view (overturn) **F** epigyne, ventral view **G** vulva, dorsal view.

Figs 1 , 2 , 3 , 5 错那侵蛛

#### Type material.

***Holotype*** ♂ (SWUC-T-TR-01-01): China, Xizang, Cona Co., Mama Township, Lebugou; 27°50′59″N, 91°46′39″E, elev. 2280 m; 4.viii.2020; L.Y. Wang, T. Yuan and Y.M. Hou leg.; ***Paratype***: 1♀(SWUC-T-TR-01-02), same data as holotype.

#### Etymology.

The epithet refers to the type locality.

#### Diagnosis.

**Figure 4.**
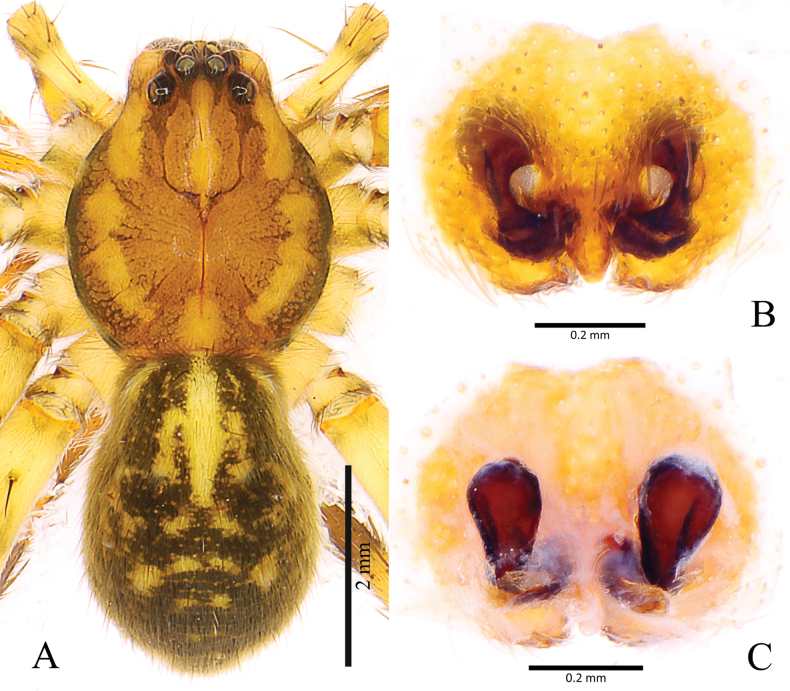
*Shinobiusorientalis* (Yaginuma, 1967) **A** female habitus, dorsal view **B** epigyne, ventral view **C** vulva, dorsal view. (courtesy of Francesco Ballarin).

The new species is similar to *S.orientalis* (Yaginuma, 1967) (Sierwald 1993: figs 20–22), but differs by having no strong spines on the male palpal tibia and cymbium (vs. present), a median apophysis with one branch (vs. two); a roundly bent and not meandering spermophor (vs. meandering) as well by having the septum of the epigyne wider posteriorly (vs. anteriorly), and slit-like copulatory openings (CO) (vs. round, cf. Fig. 2C and Fig. 4B).

#### Description.

**Male holotype** (Fig. 1A) total length 5.75. Carapace 2.85 long, 2.37 wide, cephalic part 1.8 times thinner than thoracic; opisthosoma 2.83 long, 2.59 wide. Carapace yellow brown, with distinct pattern: cephalic part behind posterior eye row light brown, anterior part of thoracic part with 2 pairs of light, and submarinal spots, larger anterior and smaller posterior; medially with thin light stripe and 2 thin, and light marginal stripes against coxa III and IV. Cervical groove indistinct, radial furrows distinct. Eye sizes and interdistances: AME 0.12, ALE 0.12, PME 0.18, PLE 0.21; AME–AME 0.13, AME–ALE 0.08, PME–PME 0.14, PME–PLE 0.23, Clypeus height 0.25. Legs yellow brown, with black pigmentation. Tibia I with four pairs of ventral spines; metatarsus I with 3 pairs of ventral spines. Tibia II with 3 pairs of ventral spines; metatarsus II with 3 pairs of ventral spines. Leg measurements: I 10.31 (2.90, 3.52, 2.50, 1.39); II 10.71 (3.03, 3.68, 2.67, 1.33); III 9.14 (2.41, 3.39, 2.21, 1.13); IV 10.84 (2.87, 3.64, 2.93, 1.40). Opisthosoma oval. Dorsum yellow brown, with black brown markings. Venter yellowish-brown.

***Palp*** (Figs 2A, B, 3A–E). Retrolateral tibial edge hood-shaped. Subtegulum large, located on baso-prolateral side of bulb. Tegulum with slanting and meandering thin spermophor. Median apophysis short, medially wide, ventrally with coracoid tip, dorsally with a groove. Conductor digitiform (longer than wide), curving and membranous. Embolus arc-shaped, bent at about right angle, with oval-shaped base (Eb), tip ends in median apophysis groove dorsally.

**Female paratype** (Fig. 1B–I) total length 5.78. Carapace 2.98 long, 2.55 wide, cephalic part 1.6 times thinner than maximal width of carapace; opisthosoma 3.03 long, 2.41 wide. Eye sizes and interdistances: AME 0.16, ALE 0.15, PME 0.24, PLE 0.23; AME–AME 0.12, AME–ALE 0.09, PME–PME 0.14, PME–PLE 0.27. Carapace pattern as in male. Clypeus height 0.15. Leg measurements: I 9.55 (2.71, 3.37, 2.29, 1.18); II 9.70 (2.80, 3.34, 2.39, 1.17); III 8.59 (2.50, 2.88, 2.19, 1.02); IV 10.54 (2.92, 3.48, 2.84, 1.30). Sternum yellowish with 3 pairs of dark round submarginal spots (Fig. 3H)

***Epigyne*** (Figs 2C, D, 3F, G). Epigynal plate 1.2 times wider than long; fovea (atrium) almost totally covered with septum, 1.2 times longer than wide, anterior part of plate 2 times thinner than posterior; copulatory openings (CO) slit-like; Endogyne with 2 pairs of receptacles, posterior receptacles (Pr) crooked; anterior receptacles (Ar) cylindrical, with the oval head covered with sparse glandular pores; Fertilization ducts arc-shaped.

#### Natural history.

Forms a funnel-shaped web on the moss. Female was found with egg-cocoons attached to spinnerets.

#### Distribution.

**Figure 5.**
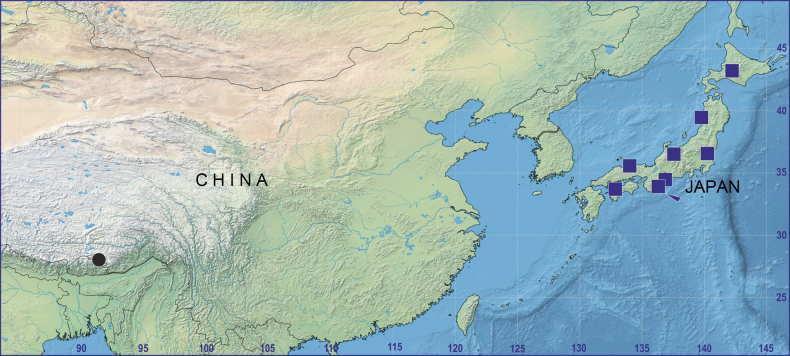
Distribution records of *Shinobius* species: *S.cona* sp. nov. (circle) and *S.orientalis* (square, type locality pointed, only prefecture records are shown).

Known only from the type locality, Xizang, China (Fig. 5).

## Supplementary Material

XML Treatment for Shinobius

XML Treatment for Shinobius cona

## ﻿Additional information

### Author ORCIDs

Lu-Yu Wang https://orcid.org/0000-0002-5250-3473

Yan-Nan Mu https://orcid.org/0000-0002-2504-673X

Feng Zhang https://orcid.org/0000-0002-3347-1031

Yuri M. Marusik https://orcid.org/0000-0002-4499-5148

Zhi-Sheng Zhang https://orcid.org/0000-0002-9304-1789

### Data availability

All of the data that support the findings of this study are available in the main text.
